# "The Hospital" Nursing Mirror

**Published:** 1898-03-05

**Authors:** 


"The Hospital" Nursing Mirror.

Being the Nursing Section of "The Hospital."

[Contributions for this Section of "The Hospital" should be addressed to the Editor, The Hospital, 28 & 29, Southampton Street, Strand, London, W.C., and should have the word "Nursing" plainly written in left-hand top corner of the envelope.]

### News from the Nursing World.

### BEDS UNVEILED AT HAMPSTEAD.

The Princess Christian visited Hampstead Hospital last Saturday, and unveiled two beds, the one in the Founder's Ward, and the other in the Harben Ward, which have been endowed from the funds raised to celebrate the Queen's Diamond Jubilee. A large number of subscribers were gathered together when the Princess arrived, accompanied by the Baroness von Egloffstein and Colonel Grant Gordon, C.B. The President of the Royal College of Physicians (Sir Samuel Wilks, Bart.), Dr. William Anderson, and Dr. Heath Strange received her, and conducted her over the building. After dedicating the beds to the poor of Hampstead, the Princess received 28 purses, containing £65, to be added to the fund now being collected to build a new hospital. A vote of thanks was proposed by Sir Samuel Wilks, and seconded by Mr. Brodie Hoare, M.P. Further donations on account of the new hospital have been received subsequent to the Princess Christian's visit, amounting to £200.

### OPENING CEREMONY AT HUCKNALL.

The Duchess of Portland opened the new Nursing Home at Hucknall which is the result of the Diamond Jubilee celebration. She received quite a royal reception, the station being decorated and the route lined with spectators, the occasion having been made a general holiday. The building, which stands in its own grounds, contains three bedrooms and a sitting-room, with committee and store-rooms and quarters for the caretaker. It is built of red brick, and the roof is tiled. The building and equipment cost £710, of which sum £60 still remains to be subscribed.

### A TELEGRAM FROM INDIA.

A telegram from India, in response to inquiries from the India Office, states they "Regret to say that Miss Harriot McDougall died of plague last night. Miss Dowson had fever on Saturday (*i.e.*, the 19th ult.), but is better." Miss H. McDougall is the youngest daughter of Mr. McDougall, of Southport, and is only 28 years of age. We sympathise sincerely with her friends in their terrible grief.

### THE NURSES' HOME AT SHOREDITCH.

Mr. Little, the chairman of the Shoreditch Board of Guardians, unveiled on the 23rd ult. the memorial tablet of the new nurses' home recently built on a site known as "The Land of Promise," adjoining the infirmary. The Home is well arranged, and the architect has shown considerable ingenuity in utilising the somewhat circumscribed area to great advantage. The lighting and ventilating have had much thought bestowed upon them. There are ventilators over each window, and the lower sash can be lifted a couple of inches without leaving any opening at the bottom. In the bedrooms additional ventilators are placed over the bedroom doors. The Home is heated throughout by hot water, and there are two fireplaces apiece in both the reading and recreation rooms. The lavatories and bathrooms are in a separate block, and can be shut off from the floors to which they belong. There are four floors. The two top ones are devoted to bedrooms, the next has the recreation-room in addition to more bedrooms, and on the ground floor there is the large comfortable reading-room, which is so arranged as to be convenient for lectures. Here there is also a linen-room. The basement is set apart for stores. The Home is comfortably furnished, and communicates with the infirmary by iron bridges. The difficulty in obtaining nurses is the chief reason for building this Home, the extra accommodation enabling the infirmary to be used as a training school, and to all the nurses employed therein the Home will, from all points of view, be a great gain.

### ANOTHER OF THE CRIMEAN NURSES.

Mrs. Wilson, who was buried at Cupar, N.B., on the 15th of last month, aged 68 years, was the second woman to land at Scutari. Married at 19 to John Fitzgerald, a private, she followed his regiment to the front when, two years later, it bore its part in the Turko-Russian War, and was, on account of her strong constitution, elected nurse. She remained until the end of the war, which left her a widow, her husband having fallen at Alma. She married a second time a comrade of his, and returned to Scotland, where the couple have for several years taken charge of the Cupar Parochial Board Hospital.

### THE LEWIS CARROL COT.

The memorial of the author of "Alice in Wonderland" is to take the form of an endowed bed at the Hospital for Sick Children at Great Ormond-street. No more suitable memento could be devised of one whose charming books take the first rank in the literature of the nursery, and fascinate alike children and their elders. All who love "Alice" may participate in the memorial by sending their contributions to the Editor of the *St. James's Gazette*.

### NURSING THE INSANE.

It is always a matter of congratulation when attention is bestowed on the training of nurses and attendants of the insane. Therefore, we read with much pleasure the communication from Dr. Macdonald, stating that for many years the County Asylum at Dorchester has granted certificates to its attendants. From a copy of the rules enclosed, we learn that for the last year attendance at the instruction given by the medical officers is compulsory. At the end of the first year an examination in elementary anatomy, physiology, and first aid is held, and certificates granted to such as have passed it. A second course, covering a further period of two years, follows, and includes lectures and practical instruction in nursing, treatment, and the care of the insane, with twelve lectures on mental disease.

Upon the candidates passing a satisfactory examination they are certified to be competent mental nurses, and are entitled to an additional wage of £2 per annum.

### TO KLONDYKE.

Some of our nurses also are on the alert to try the profitable, if uncongenial, pastures of Klondyke, and will be glad to hear that a woman has passed the famous Chilcoot Pass. Moreover, she has the honour of being the first human being to be tossed across from peak to peak on the new aërial line. The experience was a fearful one, and lasted an hour and a half. According to her own account she was strapped into a box three feet long, and two feet deep, and whirled like a bird between heaven and earth over awful precipices, to be dashed apparently against the rocks every now and again. She was cheered when she was lifted out of her circumscribed car. Her letter, dated January 31st, reached her own folk at Boston by the quickest post ever delivered from the Yukon valley. Her husband and his friends will count themselves lucky if they reach the same destination after nearly two days' hard climbing,

### DEPARTURE OF OFFICERS AND NURSES FOR AFRICA.

Late last Saturday the steamer *Bonny* left Liverpool bound for the River Forcados, Central Africa. She is laden with stores and tents, blankets, and wooden huts, and carries the detachment of officers and non-commissioned officers detailed for duty in this part of Africa some time ago. Dr. Poole and three of Guy's nurses—Miss Clarke (who is a Londoner), Miss Powell (belonging to Gerrard's Cross, Oxford), and Miss Nutt (of Oxford)—accompanied them. These will take charge of the hospitals at Lokoja, a town at some distance up the river, to reach which cargo and passengers must be trans-shipped into smaller vessels. One of the hospitals will be fitted up permanently at this place, whilst the other two are portable to be used where required.

### SARAH BERNHARDT.

Sarah Bernhardt, the celebrated actress, has successfully undergone an operation for cyst in the broad ligament at Dr. Pozzi's "Home" in the Rue d'Armaillé, Paris, and is now progressing favourably. The sufferings arising from her ailment had been aggravated by irregular meals and the strain of constant travelling. She has suffered keenly for some time, and has been carried from the stage after acting in a very nearly fainting condition.

### THE QUEEN'S NURSES IN WALES.

Cardiff is the centre for Queen Victoria's Jubilee Nurses in Wales. There are many branches, but not as yet at Swansea. The work is growing rapidly, and though the subscriptions are well maintained they do not keep pace with the increased requirements. When the association was begun in 1890 the staff consisted of a superintendent and two nurses; last year a superintendent nurse and seven nurses were working whilst the necessity of another was making itself felt. The income is between £700 and £800 a year, and is derived chiefly from subscriptions. When a new nurse is trained grants are received from the Council in London, and from the Guardians. The cost per visit averages sixpence-halfpenny. Mrs. de Courcy Hamilton has accepted the position of secretary to the council in order to permit Mrs. H. Thompson to devote her energies to the district of Cardiff alone. Needless to say that although the deficit in the income is small contributions are much needed and will be gladly received.

### LICHFIELD NURSING HOME.

The differences of opinion in the committee of the Lichfield Nursing Home have been met in a generous and sensible way by the donors of the proposed site. It will be remembered that Mr. Seckham offered a site that was not approved for several reasons. He has now withdrawn this offer, leaving it open, however, if the Building Committee should wish to reconsider it; and has, moreover, added £500 to the memorial funds of the nursing home. The Dean of Lichfield also offered a site, and he, too, is willing that his offer should remain in abeyance until the plans of the Building Committee are more matured. It is always wise to count the cost of a new undertaking, and the nursing home at Lichfield will be all the greater success because the pros and cons have been so thoroughly discussed.

### AN APPEAL.

The committee of the Nottingham and Nottinghamshire Nursing Association is making an earnest appeal through the local press for further help. The Association already employs twelve nurses and a superintendent, and there is urgent need for three more nurses. As the income last year fell short of expenditure by £92 and the cost of three nurses would be £210, the committee does not feel justified in launching into any additional expense until it has the promise of further aid.

### THE LADIES' HEALTH SOCIETY, MANCHESTER.

The Ladies' Health Society, of Manchester, is quietly doing good work in the cause of hygiene. It appoints visitors to instruct and advise the poor inhabitants in matters of health and cleanliness. Unfortunately, while some districts are fairly well off as regards these visitors, want of funds prevents the extension of the work. The annual meeting took place in the Town Hall on January 18th.

### SHORT ITEMS.

The financial difficulties of the St. John's House Nursing Association, Worcester, have been relieved by a generous gift of £500 from the late Mr. Charles Wheeley Lea, part of which has been used for current expenses, and part has been added to the endowment fund. Other donations have been received for the endowment fund, and arrangements are to be made in order to secure more systematic collections in the neighbourhood.—Why do the Southampton medical men send elsewhere for nurses when there are plenty and to spare at the Hampshire Nurses' Institute? It would be worth the committee's while to inquire closely into the apparent slight passed upon their institution, as well as to consider the chairman's suggestions with regard to advertising and affiliating the institute with a larger centre.—Miss Muriel, one of the nursing sisters who accompanied Miss Harding last spring when she went out to take charge of the Chartered Company's Hospital at Gwelo, Matabeleland, is shortly to be married to a gentleman residing in Gwelo.—Miss Pickersgill kindly informs us that she was district nurse working under the East London Nursing Society, and not a parish nurse as stated last week.

### Antiseptics for Nurses.

By a Medical Woman.

### III.—LISTER AND HIS WORK.

It is to be feared that nurses at the present time accept the antiseptic treatment of wounds, by which such splendid results are now obtained, as if it had existed from the beginning of time, and forget how much is due to that greatest of surgeons—Sir Joseph Lister.

We have already briefly traced the progress of surgical treatment, and will now shortly relate the experiments and investigations which are the foundation on which modern antiseptic treatment is built up.

The earliest experiments were conducted by Gay Lannac and others to investigate the nature of fermentation, which was found originally to be of two kinds, chemical and vital. Chemical fermentations were said to produce changes, but the ferment itself does not multiply; such is the action of pepsin or pancreatic juice.

Vital fermentations produce both change and also have the power of self-multiplication; such is alcoholic fermentation. It was decided that the changes which occur in wounds are of the nature of vital fermentation, acting slowly but steadily, and being capable of very rapid self-multiplication, with the result that putrefaction occurs, which is a form of fermentation characterised by extremely offensive odours. The next step was to discover what is the nature of this process of so called vital fermentation. Gay Lannac, Schwann, and others carried out experiments whereby they proved to their own satisfaction that fermentation was caused by the gases contained in the air, and especially oxygen, because liquids that were boiled, and from which air was subsequently excluded, did not undergo fermentation. As the result of further experiments, Liebig proved that changes only occur when a substance of the nature of a ferment is present, but that this ferment is destroyed by heat.

Later on Pasteur studied the subject of fermentation with great care, and proved that the gaseous theory was wrong, and that fermentative changes in fluids were caused entirely by the organisms contained in the air in the form of dust, and that if the air were merely left undisturbed for some time, till all the dust settled, it could then be introduced into a flask containing fluid and would produce no change. All experiments went to prove that organisms in the air cause fermentation, but not the air itself so long as it was deprived of its dust particles by heat, or passing through sulphurous acid or by filtering through cotton-wool.

It was on these experiments of Pasteur that Lister, about 1868, based his principles of the antiseptic treatment of wounds and set forth his theory as to the harmfulness of dust particles to wounds. He first proved that putrefactive changes only occurred in wounds exposed to dust particles, and that if these could be excluded no changes took place. Having proved the harmfulness of these particles, he next set to work to discover their nature, and finally convinced himself that it was organic, and consequently that dust particles could be destroyed by the same means that were used for destroying organic substances generally, and hence the origin of antiseptics or substances which prevent putrefaction.

Pasteur's experiments had shown that each particular species of organism set up a special form of fermentation outside the human body, which is the principle underlying modern bacteriology. Koch next proved indisputably that definite fermentative changes occur in wounds when specific micro organisms gain admission, of exactly the same nature as those which take place in specially prepared fluids or media. Having proved this beyond a doubt, the next step was to discover some satisfactory, thoroughly effective method of keeping wounds free from micro-organisms of every description, because it is impossible to distinguish between those that are harmless and those that are specific. For this purpose Lister introduced the spray, by means of which the air around both the wound and the operator were surrounded by a moist cloud of carbolic acid of the strength of 1 in 40, which, it was thought, would effectually clear the air of all micro-organisms. The spray was invariably used for every operation of any importance, and for dressing wounds for several years. Meanwhile further investigations were being carried on by Lister and Tyndall in this country, and by scientific men in Germany and elsewhere, with the result that it was found that the heavier particles of dust fell to the ground and left the air comparatively free. In consequence of this discovery Lister at once discontinued the spray, and admitted with the greatest candour that his theory of "air infection" was, after further investigation, a mistaken one.

Thus the theory of "air infection" was replaced by that of "contact infection," that is "the infection of a wound by contact with fine and coarse particles of all kinds, with sponges, instruments, swabs, with the surgeon's hands, and with irrigating fluids," and running side by side with this is another form of infection, namely, what may be called "implantation infection," or the infection conveyed by means of ligatures and sutures. If these are not sterilised a centre of inoculation is implanted in the centre of the wound, and all the surroundings being most favourable to multiplication and spread of these germs, they set up a most serious infection. These last are the two theories which have not yet been overturned, and on which the principles of aseptic surgery are based.

### The New and the Old Regulations at Guy's Hospital.

It is our pleasant task to congratulate the nurses and probationers of Guy's on the great improvement in the conditions under which they work. Miss Florence C. Nott Bower, the matron, has kindly sent us the chief particulars at the request of the House Committee:—

1. The trial period has been extended from one to three months, because it has been found in practice that the shorter period is insufficient to allow a sound conclusion to be formed as to the capability of each candidate, and the longer term allows a probationer who has failed in one ward to try in another.
2. The hours of duty have been curtailed, the average time now being nine hours a day, and this alteration has entailed an addition of thirty nurses to the staff.
3. Greater facilities have been afforded to each nurse to attend any place of worship she may desire. Head nurses are now allowed once in every four weeks to spend a Saturday and Sunday, or Sunday and Monday away from the hospital.
4. The day nurses have now three-quarters of an hour for dinner, instead of half an hour under the old regulations. For the night nurses a comfortable meal has been arranged shortly after midnight, which they take in the dining-hall. This change came into force last March, and we are satisfied that it has materially lessened the fatigue and exhaustion experienced by many nurses in the early hours of the morning. To carry out this improvement an addition of eight nurses to the night staff was made.

### Sick Diet: Milk.

By a Trained Nurse.

When a patient is put upon fluid-diet, the nurse's mind naturally and almost automatically reverts to milk as the staple ingredient of such a dietary. And, for the reason that milk is the sole and only food on which an invalid may live indefinitely and find efficient nourishment, it is of the utmost importance for the nurse to have an extensive acquaintance with the hundred and one forms in which milk may palatably and attractively be given. The possibilities, the drawbacks, and the limitations of milk are indeed the corner-stone of the education of the nurse-dietist.

The philosophy of milk diet as acquired in the average hospital is by no means complex or scientific. And it is no wonder with such preparation that the private nurse finds that her elementary introduction to the subtleties of sick diet begins with her first private patient. And that this is the case is unfair both to nurse and patient.

I left my training school with the belief that "milk diet" meant mugs or glasses of hot or cold milk varied by the occasional use of lime or soda water. I had read of peptonised milk in the text books, and of whey and other complexities possible to milk, but I never saw them prepared. Later, I learnt dietetics in two American hospitals and in a London children's hospital, where the utmost care is taken in the feeding of the patients. Generations of nurses go through the same experience. The eternal rice puddings and custards go on, like the brook, "for ever," and nobody pioneers a new pudding. Year after year the "pro" removes the mugs of milk—some barely tasted—from the lockers of her patients. And the "pro" promoted to the rank of staff keeps up the custom. I propose to deal with milk diet from a totally different point of view. And I hope to show that milk may be varied to a marvellous degree, and may be presented in so many forms that no invalid should ever have to complain of monotony.

The value of milk as an article of food has been taught us with our alphabet. But we are often told in nursing and health books that milk is "most easily digested," a statement which has many reservations and exceptions. For undiluted milk is by no means well digested by the average adult, and much harm is done by the deep-rooted belief that milk is a "natural food," and can therefore be easily disposed of by all and sundry. Many a nurse labours under the impression that because the invalid under her care is taking large quantities of milk he is on the safe side of nutrition. "He is taking plenty of milk," she says, "so he must be all right." But this by no means follows. Many a typhoid patient absolutely starves in the midst of a plentiful milk diet just because this is not given in a form which he can assimilate. Milk is the traditional food of typhoids, but in four cases out of five it needs very special preparation to accommodate it to the disturbed digestive conditions present in more or less degree in this disease.

When an adult patient is put "on milk," one of the first difficulties the nurse has to encounter is the instinctive dislike the average "grown up" evinces towards a return to the fluid of the nursery. For milk is essentially a food for babies rather than for adults. However strong the argument in favour of milk as a nutritive drink may be, there is no question that it is not a natural food for the adult, for milk does not go well with the nitrogenous and mixed dietary of every-day life. It is too concentrated a form of nourishment to be needed by persons living on the good ordinary diet of meat, eggs, fish, &c., commonly taken. It must have occurred to many nurses that milk is more readily taken and digested by patients who have been accustomed to eat very little meat. In an Arcadian and vegetarian state of society, milk, doubtless, would be eminently suited to all. But this is an epoch of high and complex feeding, and a return to "baby food" during illness presents many difficulties. The change on the part of persons long accustomed to highly-seasoned and spiced dishes to a *régime* of bland, unappetising milk foods is an undoubted trial; and unquestionably to this must be attributed in large measure the want of appetite for and the inability to digest easily such an unaccustomed diet. Perhaps because this fact is receiving recognition, sick-room feeding is tending towards more flavourings and appetite-stimulating factors in invalid diet. And we are beginning to understand that the cookery of the sick must be differentiated to varying circumstances. For instance, the club man accustomed to game and richly-flavoured foods, will need more beef-teas and meaty infusions introduced into his fluid diet than will a delicate woman with little appetite who has long been accustomed to a tea and cake regimen. Which leads us to the moral that it is the individual we feed, and not the disease. Where milk enters largely into a fluid diet, it becomes of the utmost importance that the milk given should be fresh, pure, and good, and this question will be discussed in the next article.

### Appointments.

### MATRONS.

Northamptonshire Institute for Trained Nurses.—On February 18th Miss Annie T. Lunn was appointed Lady Superintendent of this institute. She was trained at the Macclesfield General Infirmary, and at the Metropolitan Nursing Association, Bloomsbury (the Central Home for training Q.V.J.N.). She has held appointments as assistant nurse West London Hospital and extra staff nurse at St. Thomas's. She has been Queen's nurse at Northampton and at Chertsey (a branch of H.R.H. Princess Christian's Nurses' Home), and has had charge of the home and organisation of the district nursing at Huddersfield.

Borough of Bootle Corporation Hospital.—Miss Martha Johnson, who has for the past four years been sister of the male surgical wards of the Bootle Borough Hospital, was elected Matron of the same on January 31st. She was trained at the Hospital for Women, Shaw Street, Liverpool, and has had experience in fever nursing in the City Hospital, Netherfield Road, Liverpool, and at the Eastern Hospital, Homerton, London. She has also been charge nurse Bucks General Infirmary at Aylesbury.

Brymbo (near Wrexham) District Jubilee Nursing Association.—Miss Gladwys Jones, who was trained at the Royal Infirmary, Liverpool, and subsequently worked in a Liverpool District as a Queen's nurse, was appointed District Nurse for this Association on January 26th, and commenced duties on February 14th, 1898.

Great Yarmouth Workhouse.—Miss Emilie Jane Ablitt was appointed Superintendent Nurse on February 25th. She was trained at Glasgow Western Infirmary, was afterwards charge nurse at Shoreditch Infirmary, and for the last seven years has been head nurse of the Greenwich Union Infirmary.

### Minor Appointments.

Great Yarmouth Workhouse.—On January 28th Miss Ellen Charlotte Day was appointed Charge Nurse of this workhouse. She was trained and afterwards became assistant nurse at Hackney Infirmary. She was charge nurse at Chertsey Infirmary until the December of last year.

South-Western Fever Hospital, Stockwell, S.W.—Miss Grace Mackenzie, who was trained at the Edinburgh Royal Infirmary, has been appointed Charge Nurse of this hospital.

### Nursing in Paris Hospitals.

### A.—LAY NURSES.

### IV.—SEX, CONDITION, AND CHARACTER.

THE modern Paris hospital nurses contrast in many ways with those of London, among other things in the matter of sex, condition, and general character. On July 1st, 1896, of the over 5,000 nurses in all the hospitals and asylums under the Assistance Publique more than 2,000 were males, the exact figures being 2,968 women to 2,148 men, or 5,116 in all. It is necessary to note, however, that some 400 of the male nurses were acting as details for other duties than work in the hospital wards. Again, as to the condition, not only are a large proportion of the male nurses married, but also a considerable number of the female nurses.

There is comparatively little difference in the source of supply of nurses of either sex. The applicants have been almost always workpeople out of place, the larger number being household servants, and, next to them, shop assistants. It is no special aptitude for nursing nor enthusiasm for a career of beneficence to humanity which leads these applicants to the hospital gates; it is simply bare necessity. The class of devotees from well-to-do and cultured circles which is such a feature of English hospitals figures in Paris hospitals to a very small extent.

Naturally, the nurses enlisted in this fashion have not hitherto been, as a whole, of the most desirable character. Far be it from me to question the fact that the bulk of them have performed their duty to the best of their abilities. There have been, especially in the earlier years of lay nursing, some scandals concerning negligence and incompetence, and even dishonesty—that meanest of all dishonesty, the robbing of the helpless; but, as a whole, the nurses have been fairly creditable. Nevertheless, their capacity has been naturally in many respects defective. While few of the women are skilled in any employment but household affairs, very few of the men are of the artisan class. Such of the men as are artisans mostly belong to a very undesirable element in the recruitment of nurses; these are employed in trades which are intermittent in employment, or persons waiting for places in the public service, like the police or post office. They get the tip from some fellow-workman to apply at a certain hospital, only to leave as soon as some other employment is obtainable. This nuisance for the hospital administration is not very much an element in the case of the women applicants. Many of the applicants are married when they enter the service. In the case of men, of course, this is not of any moment; but with the women the question of marriage is the subject of particular investigation. As the chief of one hospital remarked to me, the Parisian hospital directors do not love Parisian husbands for their women nurses. The country applicants, whose husbands remain in the country, are much to be preferred. If the husband is in Paris, he may at any time assert his authority, to the detriment of hospital discipline, or make himself a nuisance in many ways by seducing his spouse from her duty, even if he does not use more arbitrary methods.

Of course, there are a large number of married couples, with both spouses in the hospital service. They are evidently the most convenient and natural of married nurses. They form, however, only a small minority; and there is always the vexed question of relations of the women nurses with their husbands and children. I have inquired particularly of all the directors as to the relative qualifications and fidelity of married and single women, and most of them declare that there is no choice so far as the patients are concerned. The directors of two of the largest hospitals, although acknowledging the equal willingness of the married nurses, were in favour of celibacy. In fact, one director told me that if he only had the power he would tell a nurse who wanted to marry to leave the hospital service and attend to her husband. The other director said that the ideal nurse would be an orphan with no family to bother the administration. Some few, however, discriminated in favour of the married nurses. M. Mulheim, at Saint Antoine, thinks married nurses more serious and experienced; M. Valet, at the Trosseau Hospital, says there is more guarantee with married than with the single; and M. Baron, at the Cochin Hospital, says he has found the married nurses the most devoted of all, especially those with children, and that the prospective mothers are particularly faithful and able up to the day of their lying-in. The subject of small pilferings and leaking in hospital stores among the families of married nurses I will touch upon when dealing with wage questions.

M. Baron is on every hand a partisan of women as nurses. There are in his establishment but few men, and these for heavy tasks, and he wants no more. He says that in character, temperament, and fidelity women are far more qualified for nursing than men. Although this opinion has been the traditional idea of the world in general from earliest ages, it is not entirely accepted by all the chiefs of the Paris hospital service. I have found considerable diversity of opinion. Some assert that the men are, as a class, far more imbued with the professional spirit, while the women only enter the hospital as a makeshift. This is by no means the idea of the committee appointed by the Assistance Publique in 1896 to study the question of diseases occurring among nurses during the great tuberculosis scare. In their report they incidentally remark that the women nurses are as a rule satisfactory to their chiefs. The male nurses, on the other hand, the committee remarks, have often been recruited from ex-patients, only partially cured, who were still suffering from serious maladies, such as tuberculosis, and that they are accepted by the Assistance Publique as much through pity as necessity. In at least one hospital the engagement of ex-patients as nurses is almost the rule rather than the exception. This is at the Saint Louis Hospital, where the whole establishment is permeated with the often horribly disfigured mementos of previous terrible ravages and suffering. The excuse for this is that no one outside the wards of Saint Louis is willing to give these hapless beings employment, and common charity requires their being given situations by preference when they are able to perform the tasks set them. Perhaps, however, another and less benevolent reason is at work. It is possibly not always easy to recruit from outside efficient help for the repulsive work of a skin disease hospital like St. Louis.

It is almost certain that these unfortunates at Saint Louis will ere long have to make way for more desirable custodians and be themselves assigned to some kindly retreat. The battle cry of the hospital reformers in Paris just now is a demand for absolutely healthy nurses. The newly constituted medical examination of candidates is only a first step, but it is not only tuberculosis in nurses which is to be tabooed, but physical deficiencies of all sorts.

The best nurses are said to be the Breton women, who until recent years formed by far the largest element. In this, as in other things, Brittany seems to be the salvation of modern France, as the fecundity of the same Breton women keeps the depopulation of France from being too glaring. This also seems to indicate that motherhood and the nursing faculty go together; but, then, the Bretons are also the most religious, and furnish the greatest contingent of celibate sisters. It seems to me that not only Breton women, but the women generally come out of the trial far ahead of the men, in spite of the advocates to the contrary. It is significant that where male nurses are employed there is always a woman matron nurse to superintend each ward. One of the chief authorities of the administration says that two-thirds of the men are willing to leave the service at any moment for another job, and the other third only stay for promotion or advancement, and that the women are by far the best nurses. The proportion of men has been decreasing for 25 years past, and there seems little reason to believe that the project favoured by many of having all nursing of male patients done by male nurses, leaving women to women, will ever be realised. Some directors advocate men for giving baths, for errands, and various incidental tasks, but for actual work in the wards the consensus of opinion is overwhelmingly in favour of the gentler sex.

EDMUND R. SPEARMAN.

### The Children's Nurse.

THE Queen and six of her children have united in raising a memorial to Mrs. Thurston, who was nurse to the Royal Family from 1845 to 1867, and who is buried in Kensal Green Cemetery. The Princess Louise herself designed the monument. It is gray marble wheel cross mounted on slabs, decreasing in size. Where the arms of the cross join is a bas relief in white marble, representing a nurse shielding two children from a storm under her cloak, and the plinth bears this inscription: "In grateful and loving memory of Mary Ann Thurston, by V.R. and I. and her children, Victoria, Albert Edward, Alfred, Helena Louise, Arthur, Beatrice."

It is not uncommon to hear those who have arrived at the dignity of a certificate speak of the children's nurse with a considerable degree of condescension. This attitude of mind is ungenerous, and not a little short-sighted. There is nothing derogatory in honest work, and the care of the child is inherently as honourable a task as the care of the sick. The only difference is that, thanks to the inspiration of Florence Nightingale and others following in her wake, nursing has been rescued from the sphere of the unskilled hireling; and as a higher and still a higher standard, both of work and workers, is demanded, so the status of the profession and those following it constantly rises. What has been done for the sick is still to be done for the child, and, sooner or later, it will be done to the gain alike of the children, the parents, and the vast army of educated women who must learn to labour truly to earn their own living. Unskilled labour is in the greatest degree extravagant, and is low priced because it is practically worthless. Taking nursing as an example, Mrs. Gamp and her kind would receive more kicks than halfpence at the present time, and the quality of their work may be judged when it is found cheaper to engage skilled nurses to attend to paupers than to trust them to the tender mercies of women of their own class. Let it be observed the trained nurse is not superseding the pauper solely from the humanitarian point of view, but because it is commercially cheaper to employ her. The majority of children's nurses are untrained, and they are, therefore, as a rule, dear at any price. Careful mothers, who can afford it, pay long prices to obtain experienced nurses; those who cannot afford it keep their children with themselves as much as possible. Careless mothers are horrified when they discover too late how much mischief has been done to their children's minds and hearts by contact with a low-toned if not actually immoral ignorance. Educated women alone are fit to be entrusted with the momentous task of forming the characters of the young.

Already the demand for trained nurses in the nursery is making itself felt, and the question asked by those who have to do with this branch of labour is why should not trained nurses whose strength is not equal to sick nursing enter into this comparatively new field, conquering it for good as they have conquered the sick room. The truth of the matter is they consider it *infra dig*. But surely there is nothing to jar the most refined in the environment of a well-ordered nursery. As it is undesirable that any nurse should mix freely with the other servants, a lady would still be able to choose her own associates and to possess a bed-room of her own. Whatever there may be lost in regard to position would speedily be dispelled when once the work is taken up by the right kind of woman. And it will be taken up. The care of children is more essentially a woman's work than even the care of the sick. It requires patience, tact, trained common sense, wholesome affection, high principle, and refinement; but it does not require brilliant mental attainments. There are plenty of ladies in every sense of the term who have neither the qualifications nor the desire to shine in intellectual pursuits, but who would be entirely desirable as the guardian angels of our little ones.

Nor are the rewards of the calling to be despised. Possibly Mrs. Thurston, on whose grave stands a monument bearing some of the highest names on earth, lived in closer fellowship with our royal house than any other person outside that family circle. She was knit to them and they to her by a thousand ties that the world knows not of, but so strong, so deep, that her nurslings have seen fit to record their appreciation of herself and her services in the sight of all. When such is the case it is only to be supposed that adequate pecuniary reward is not lacking.

Nurses, the springtide of whose strength is passing, is it not worth while to enter this kindred profession not less honourable but less arduous, for which your training renders you so fit—to be the pioneers of the army who will rescue this fair province of woman's work from the rule of the ignorant?

### Wants and Workers.

MISS THOMPSON, 18, Holland Road, Brighton, wishes to have the address of May Williams, formerly of Ipswich

### Nursing the Plague.

By a Sister on Duty.

Having been successful in obtaining the appointment of nurse for "plague" duty in India, I received less than one week's notice of departure, so, as may be imagined, my preparations had to be somewhat hurried, and as so many kind friends suggested my getting so many different things, I was somewhat nonplussed as to what I should really require. It may here be stated for the sake of any other nurses coming out in a similar way, that only summer clothing is needed. A cashmere and alpaca coat and skirt, some smart blouses and shirts, summer hats, a couple of evening dresses, some nice shoes and gloves, the latter being very expensive and difficult to procure in India, are most suitable. Anything in the way of uniform can easily be made in India, as the native tailors can copy a pattern most accurately, and charge very little.

Nurses on "plague" duty wear the Indian Nursing Service uniform, which is white, with red collars, cuffs, and belt. A grey cloak and bonnet are useful, though not absolutely necessary. I received £10 as outfit allowance, together with a first-class railway ticket for the journey from Victoria Station to Brindisi; and after procuring what I hoped were suitable things for Indian nursing, the time very soon came to say "good-bye," and I, with ten other nurses, left Victoria Station *en route* for Brindisi.

As regards the journey there is nothing much to relate; it was very amusing, and not altogether comfortable. And here it may again be added for the sake of any future emigrants, that nurses should be well supplied with cash, the charge for excess luggage must be paid, the small allowance of sixty-five pounds being practically nothing. Nurses taking their luggage across the Continent must be prepared to pay between £4 and £5. Then the question of food must also be considered for a journey of two days, and as we arrived in Brindisi a day before the departure of the ship, we had the additional expense of a hotel bill. I do not mean to convey that the expenses incurred by the nurses are not refunded to them; on the contrary, we were told to send in our claims to the Indian Government immediately, and that we should be repaid, but as this must of necessity take some little time, nurses should be careful to provide themselves with a fair amount of ready money before undertaking such a journey.

We were allowed second-saloon passages in a P. and O. boat. The voyage was very interesting, and, to many nurses who had never travelled abroad, it was most enjoyable. We arrived in Bombay early in December, and found it very hot. We were charmed with the fine city, and as we were allowed to spend a few days there in order to do some necessary shopping, for which we all received one month's salary in advance, we took the opportunity of seeing as much as possible.

After a few days our party was broken up, and five of us proceeded to Poona, where there is the largest "plague" camp in India. The medical staff consists of one supervising medical officer, one lady doctor, two doctors, one of the latter residing in the camp, and about ten native medical assistants. The nursing staff consists of fifteen English certificated nurses. The plague hospital is built in the form of long low sheds made of rough matting nailed together with bamboo, and painted white. The floors are of mud; the beds are of rough wood, with rope laced across; each patient has one sheet, one pillow, and three blankets; and, owing to the exertions of some of the medical staff and of the kind assistance of friends in Poona, all the patients have been provided with bed jackets, special colours having been chosen for the several wards. These have been a great improvement, both as regards comfort and cleanliness. The rest of the furniture of the shed consists of a wooden box for sheets and blankets, and a tin dressing-box.

And now for a few words on the nursing of "plague." Things have improved so much in this hospital that one almost forgets how hard it was at first. When we began work here, each sister had charge of five or six wards containing in all about sixty patients, and as we have only ayahs and ward boys to help us, who have no idea of nursing, or even of making a bed, the work seemed very hard. Not only had we to contend with the ignorance of the natives, but also with their strange customs and prejudices; but as the staff of nurses increased we had more time to teach the native attendants, and to look more carefully into the actual details of the nursing, feeding, and care of the patients. It seemed almost hopeless at first to make the ayahs and ward boys understand that on no account were the patients to be allowed to sit up, and many instances have been known when patients have died solely through this neglect. The death-rate has been enormous, and many a time there have been four and five deaths a day in the ward, and it was quite a common thing for the nurses on night duty to find many of the bad cases dead during their round.

The common symptoms of plague are high temperature, delirium, staggering gait, loss of speech, indifference to surroundings, frontal headache, an anxious look, a furred tongue with clean tip and sides and injected conjunctiva; the patient usually has a bubo. On admission, if the patient has these symptoms, the usual treatment, I may briefly say, consists in trying to reduce the temperature—the application of an ice bag to the head and hot-water bottles to the feet. Sometimes a hot pack is effectual; the patient needs a good deal of stimulating. If necessary, the bubo is incised and well irrigated with tincture of iodine lotion and poulticed. Septic pneumonia is a common complication and very often a fatal one. As regards diet, the patients, until they are convalescent, take milk, sago, and raw eggs beaten up.

The hours on and off duty are as follows: We are called at six a.m. and have our breakfast or "chota-hazri" brought to us. This consists of coffee, eggs, and buttered toast. We are on duty at seven a.m. and immediately see to the making of the beds, taking of temperature, pulse, and respiration, and general supervision. We are very busy until twelve o'clock, the doctors' first visits to the wards being from nine until twelve o'clock, after which time we go off duty, and very glad we are to retire to our bungalows after five hours' hard work, which not only consists in doing as much as possible in as short a time as possible, but in walking about from shed to shed in the burning sun, which even at this time of the year we find very trying. We are obliged to wear "topees" all day and carry double-lined umbrellas. We breakfast at half-past twelve, then half the nurses go on duty again from half past one until four p.m., and the other half from four until seven p.m. We dine at eight p.m., and most of us retire to bed soon after.

The night nurses, three in number, go on duty at eight p.m. and take entire charge of the camp, a medical officer and native assistants sleeping on the premises. All the sheds must be visited at least three times, and the bad cases as frequently as possible. I must confess night duty is somewhat gruesome; each sister goes about the camp carrying a lantern, and I think we all have a natural dread of entering those dimly-lighted sheds, containing several bad cases, and not quite knowing how many we should find alive since we last saw them. As regards the nursing at night, we do as much as possible for the bad cases, but our visits have to be brief, as there are so many who need attention. The howling of the dogs and jackals is most dismal, and we have also seen an additional horror in the shape of a snake. The nights are cold, and when we return to our own private shed after our round of the camp we are very glad to gather round the "sigaree," which I may explain for the benefit of my English readers means a three-legged charcoal burner deposited on a sheet of iron and which requires constant fanning to keep alive; the result of the fanning is that you get very cold yourself and succeed in smothering your companions with ashes. Our nightly cooking is also conducted on this primitive arrangement.

The improvements in this camp have been very great, and everything has been done for the comfort and welfare of our patients. We all consider ourselves most fortunate in having worked here, which is not only the largest but the best managed plague camp in India.

In conclusion, I should like to add a few words as regards the pecuniary benefit to be derived by our present appointment. Our salary is 175 rupees per month, and our expenses, including servants, food, deductions for income tax, small subscriptions, &c., should not exceed Rs. 75 a month; the remainder is, I consider, quite sufficient, not only for dress, carriage hire, amusements, &c., but enough to enable every nurse to save a certain amount quite easily every month. The expenses in some parts of India are, I believe, greater than in others; but, judging from what our monthly expenses are in Poona, I certainly consider that every nurse, unless she is very extravagant, ought to be able to save a little of her income. Those who have bicycles are very fortunate, for carriage hire runs away with a good deal of money. The Indian climate is not suitable for walking exercise, especially after a hard day's nursing in the camp, and the dusty roads are not inviting, so driving is a necessary luxury.

### On the Employment of Trained Nurses in Workhouses.

On the 2nd inst. Dr. John Milson Rhodes, M.D., chairman of the Chorlton Union, read an excellent paper at the Poor Law Conference held in London on the employment of trained nurses in workhouses. Dr. Rhodes, in comparing the number of sick and infirm in the voluntary hospitals and those supported by the rates, showed the preponderance of paupers to be tremendous, the actual numbers in the voluntary hospitals being 16,400, whilst in the so-called sick wards of our workhouses there are no fewer than 39,264 sick and bedridden, with an additional 39,264 aged and infirm, amounting to a total of nearly 60,000. He said the problem of nursing this army of sick was not a simple matter. The more it was studied the more apparent became the difficulties attending it, difficulties which were not altogether solved by the employment of trained nurses. Large centres like Liverpool, Birmingham, and Chorlton could run their establishments on the lines of first-class hospitals, but it was far otherwise in country districts. There were at least thirty counties in England and Wales the total number of the sick in whose workhouses was less than those in one of the infirmaries just mentioned. Lunesdale, for instance, has no sick, no infirm, no bedridden, and no aged. Stratton has no sick and no bedridden, whilst Aysgarth, in Yorkshire, has only one aged and infirm. One county in Wales has four separate workhouses, the sick in which number 23 and the aged and infirm 18. In the opinion of Dr. Rhodes the solution is for a number of unions to combine and fully equip a few hospitals, as is done for epileptics, imbeciles, and vagrants. The difficulties of transit to the patients are not great with railways and rubber-tyred ambulances, whilst the plea against removing patients so far from their friends is non-existent. The books of any large town hospital will show that many of the inmates have come long distances for treatment. Dr. Rhodes adopts the same threefold classification as Mr. Baldwin Fleming in his paper on "Workhouse Nursing" reading before the Hampshire Guardians' Association—a digest of which paper is in the "Mirror" for December 12th: First, those requiring a permanent staff large enough for all occasions; second, those with a trained nurse and employing extra help when needed; and third, those which only sometimes want the services of a nurse. Classes 2 and 3 offer the greatest difficulties with regard to the nursing question. From a variety of causes service in workhouses is not attractive to nurses. The cases to be nursed are not, as a rule, interesting, being of a chronic and senile character, and often revolting in themselves. The workhouses, too, are built where land is cheap, and therefore in the country, and often walled round so that they resemble prisons. Nor is the accommodation always satisfactory to the nurses, whatever it may be to the Guardians. A separate bedroom and a sitting-room furnished with low easy chairs only afford rest and comfort, to which nurses have a right when they come off duty tired. Nurses also require regular hours of relaxation. A couple of hours daily in the open air is not too much for those who have been attending, perhaps, to repulsive sores. Half a day once a week, a whole day once a month, and three weeks' holiday in the year, is the scale approved by Dr. Rhodes. The diet, too, ought to be more varied. One day in the week ought to see the perpetual mutton and beef banished from the nurses' table. The steward will have a little extra trouble in arranging it, but he will have less in other ways. Dr. Rhodes points out that there is a mental side of the diet question, and asks his hearers to consider the effect the announcement "that there would be nothing but cold mutton for dinner would have on their temper," and warns them that "they would very soon find that nothing disturbs their equanimity of mind more than a bad diet." The food should be good, of liberal quantity, and well cooked. With regard to the cooking, Dr. Rhodes thinks that much might be done if ladies would take the matter in hand and establish schools for training in workhouse cookery, as there are for ships' cooks and others. The cooking and nursing appliances also ought to be better; and waxed floors, spring mattrasses, and bed-lifts are necessities. The question of night nursing is very important. To require a nurse to do night duty continuously for months is not conducive to health, and one month in three is the maximum that ought to be expected. Night nurses must also be well fed, and their sleeping apartments should be away from the noise and bustle of the house. If it is impossible to isolate them, at any rate the Guardians can have the floors near their rooms covered with cork carpet or matting. The number of nurses is always a difficulty, as there can be no definite rule on the subject. Three nurses to 45 is not too many with a large percentage of chronic cases; that is, one night and two day nurses. Dr. Rhodes thinks that the salaries should be more generous; it will be found, he says, true economy to add to the wages the £5 so often spent in changing officers. By obtaining good nurses and treating them well, the real origin of many scandals—often of much more importance than the scandals themselves—would be removed. From the hundreds of letters on the subject that Dr. Rhodes has received he is convinced that, given a good Board of Guardians, a good hospital-trained matron, and a good staff of nurses the patients will be so treated as to make them more useful members of society. They will also be taught something of the necessity of light and air and cleanliness, and thus, perhaps, some of those insanitary causes to which so much sickness is due may be removed.

### Everybody's Opinion.

[Correspondence on all subjects is invited, but we cannot in any way be responsible for the opinions expressed by our correspondents. No communication can be entertained if the name and address of the correspondent is not given, as a guarantee of good faith but not necessarily for publication, or unless one side of the paper only is written on.]

### TRIALS OF A DISTRICT NURSE.

"ROTUNDA" writes: It would be impossible to enter into the objections to combining general and midwifery nursing in one person. It is never satisfactory and always risky; no nurse ought to undertake both. As a district midwife I generally follow this rule. I leave my patient as soon as she is comfortable, and the baby washed and dressed, always leaving behind some well-cooked gruel and a list of my visits, in order, so as to enable people to send for me if wanted. I return and see her in four hours' time, and again eight hours after the birth. After that I visit her twice daily, washing and dressing the infant, until the cord is off and both are doing well. Then once a day till I see her about again. There is generally a neighbour or handy woman about the house who will see my orders are carried out, and they are always glad to be of use. It is unreasonable to expect more from a busy parish nurse. Like a doctor, a midwife must be prompt to attend a case when called, night or day, and in difficult labour may have to spend several hours in the house; therefore she must secure her rest when she can, but secure it she must for her own sake and the sake of those she is nursing. If she is a general nurse this must often be impossible.

### THE PLAISTOW CHARITY—THE HOLT-OCKLEY SYSTEM.

CONSTANCE COCHRANE writes: In reference to your article in the "Nursing Mirror" of February 5th on the Plaistow Charity and the Holt-Ockley system, I beg to enclose a short account of a branch of the latter association which has been recently started in Cambridgeshire, and I would also like to add that neither of our nurses have taken their L.O.S. certificates, as the advice at the head office of the Holt-Ockley Association was distinctly averse to their being allowed to attempt it, and each nurse at the commencement of her training is furnished with the following notice: "You are asked to take notice that your training as a monthly nurse at Plaistow will not fit you to undertake midwifery work. And you are, therefore, recommended not to make a practice of attending confinement cases unless the attendance of a doctor or of a qualified midwife is arranged for." I am aware that many persons object to the methods of the Holt-Ockley system, but can they offer to supply us in any other way with nurses for small scattered country villages with populations varying from 150 to 500, and consisting almost entirely of farmers and agricultural labourers? Most of the few resident gentry already subscribe to the hospitals, and have many other local calls on their purses, not very overflowing in corn-growing districts, and the £5 to £8 guarantee is as much as they can promise annually for each village. With a population of three hundred the subscriptions from the poor people and farmers will come to about £2 10s. a-year. Surely half a loaf is better than no bread at all. Until last October, when our cottagers were ill they could oftener than not get no one at all to nurse them, except perhaps, an eleven-year-old daughter, or a son out of work, or a deaf old woman of seventy, or a neighbour to look in occasionally for a few moments to make a cup of tea. Is it of no advantage to be tended by a capable woman who has a real liking for the work—for the inducements are not sufficiently large to tempt others—and who can cook nice little dishes, take temperatures and pulse, administer enemas, change sheets, prepare poultices and fomentations, and perform many other little offices of help and comfort that can be easily learned by a woman of ordinary intelligence in six months. Why are the short courses of lectures on "Home Nursing," given by the St. John Ambulance Association and the National Health Society, so largely encouraged and approved by all classes? And yet when the same instruction is given in a practical form for six months there is an outcry because it is not longer. The wife of a labourer earning 11s. a week (the wages now paid in Cambridgeshire villages) cannot afford to feed a nurse and a charwoman as well, and she would not receive into her house any other than a homely woman who had been used to cottage fare and could manage to cook in the single saucepan, eat no other meat than pork, and make shift with the scantiest possible supply of sheets. The Lady Bountiful of fiction does not by any means always exist in remote districts, and the distance between the villages would in any case make it impossible for a nurse to return to her home for meals. It is only for country villages, and not for towns, that the promoters of the Holt-Ockley system recommend their scheme. For towns they consider that district nurses are decidedly the best. These of course can be as amply trained and paid as the funds of the district will allow. In conclusion I would add that the Holt-Ockley system has lately received great assistance and encouragement from St. John Ambulance Association, whose action in the matter has been countenanced and approved by his Royal Highness the Prince of Wales.

### The Book World for Women and Nurses.

[We invite Correspondence, Criticism, Enquiries, and Notes on Books likely to interest Women and Nurses. Address, Editor, THE HOSPITAL (Nurses' Book World), 28 & 29, Southampton Street, Strand, London, W.C.]

### CASSELL'S ILLUSTRATED HISTORY OF ENGLAND.

Messrs Cassell and Co., with their wonted spirit of enterprise, are publishing a profusely-illustrated English history, which is to appear weekly for the price of 6d. a copy, and the history will be completed in 53 weekly parts. Through this means in a year's time those who are forgetting their history will have a very pleasant means of retouching their memories, and this weekly volume will be looked forward to both for its excellent letterpress and interesting illustrations. Part I., now before us for review, commences with an account of the Roman rule in Britain, and the two frontispieces are "King John Signing the Magna Charta" and "The Landing of the Romans on the Coast of Kent." A coupon will be attached to Parts I. to IV., which, if cut out and forwarded to the publishers, entitles the subscriber to a copy of the large picture of "The Thanksgiving Service at St. Paul's Cathedral" for the nominal sum of 6d.

### Presentations.

A DEPUTATION of "old pupils" called at the British Hospital on February 28th to say good-by to Miss Freeman, who has resigned the matronship after thirty years' work for the hospital. Miss Freeman was asked by the "old pupils" present to accept a little remembrance of the happy days they had spent there under her tuition in the form of a silver cake dish, which bears the following inscription: "To Miss Freeman, with the affection and respect of many old pupils." The list of "old pupils," which is not yet complete, will be sent to Miss Freeman later.

MISS PHILLIPS, on resigning the Matronship of the Norwood Hospital, which appointment she held for more than fifteen years, was the recipient of several gifts, namely, an engraved salver with inscription, from nurses and servants; opal and diamond ring, subscribed for by friends; gold brooch and bracelet, album, purse, &c.; and, just to hand, a cheque for fifty guineas as a public testimonial.

### Where to Go.

ROYAL BRITISH NURSES' ASSOCIATION.—Miss Mary Kingsley will deliver the third sessional lecture on Tuesday, March 8th, at 11, Chandos Street, Cavendish Square. The subject will be "West Africa." The chair will be taken at eight p.m. by Mrs. Dacre Craven.

### For Reading to the Sick.

"PERFECT through suffering."—*Heb.* ii. 10.

**Verses.**

Let not my years of weakness all be waste,
But, like the fallow field,
A preparation and refreshment, too,
For richer harvest yield.

Yea, Thou cans't make this Vale of Bacca drear,
A well of water pure;
The streams whereof are fed from out Thy Throne,
And will for aye endure.

Thou art my Father, and Thou knowest best
Thy Purpose and my skill;
My times and seasons all to Thee belong;
Perfect me in Thy Will. —*S. Scott.*

O Word of words, O Name of names,
All other names above;
O Song of songs, O Joy of joys,
The great Keynote of love.

O rest for all our weariness,
O balm for every pain,
Healing of all our sicknesses.
And cleansing for each stain. —*H. J. Evans.*

Suffering, I came to Jesus,
Wounded and sick within,
With Nature's leper plague scourge,
The dread disease of sin;
Oh! if Thou wilt, I pleaded,
Lord, Thou cans't make me whole,
And He, with oil of healing,
With joy has filled my soul. —*H. J. E.*

Pain, that to us mortal clings,
Is but the pushing of our wings,
That we have no use of yet,
And the uprooting of our feet
From the soil where they are set,
And the land we reckon sweet.
—*J. Ingelow.*

Grant a cause to remove, grant an end to attain,
Grant both to be just,—and what mercy in pain.
—*Lytton.*

**Reading.**

"In weariness and painfulness, in watchings often."—2 *Cor.* xi. 27.

St. Paul probably did not mean exactly what I am suffering now—the weariness and painfulness of a sick bed, the sleeplessness arising from illness. Most likely he meant the weariness and painfulness caused by toilsome journeys and hard labour and occasional want; and his watchings were probably when he was labouring and travelling by night, instead of resting on a bed. And though my circumstances are different from his, yet the words of the Apostle exactly describe my state. I, too, am "in weariness and painfulness." I, too, often watch through the greater part of the night, tossing restlessly, unable to sleep. I may learn a lesson from the Apostle. In a long list of trials and sufferings, how much there is that was painful to flesh and blood! Naturally, he no more loved hardship and discomfort than I do. Yet, far from complaining, he even glorified in the things which concerned his infirmities in the privations and sufferings which so dear and gracious a Master called him to endure for His sake; and willingly went forward, still in the same course. Help me to bring honour to Thee by meekness and patience. Teach me to count it all joy when I fall into tribulation.—*Author of "The Week of Mourning."*

### Notes and Queries.

**The contents of the Editor's Letter-box have now reached such unwieldy proportions that it has become necessary to establish a hard and fast rule regarding Answers to Correspondents. In future, all questions requiring replies will continue to be answered in this column without any fee. If an answer is required by letter, a fee of half-a-crown must be enclosed with the note containing the enquiry. We are always pleased to help our numerous correspondents to the fullest extent, and we can trust them to sympathise in the overwhelming amount of writing which makes the new rules a necessity. Every communication must be accompanied by the writer's name and address, otherwise it will receive no attention.**

*Nursing Abroad.*

(184) Can any nurse give me information respecting English nurses who are engaged in private nursing abroad. I intend to go to Paris the latter part of this year, from there to Nice, and on to Italy. Is uniform (indoor and out) worn as in England? I am a certificated medical and surgical nurse, and intend to take up either monthly work or massage, whichever I am advised is most likely to succeed.—*Jenny.*

You must be most careful in making arrangements to travel abroad. Unless you go out to a reliable case, would it not be better to go at first under the auspices of an in titution such as the Hollond Institute, whose head-quarters are 1, Tavistock Chambers, Bloomsbury, W., and which has branches in the places named? The salary is not high at first, but there is no danger of one of its nurses being turned out penniless at a moment's notice.

*Ste ardess.*

(185) I want a post as stewardess on one of the P. and O. boats. Is it necessary to be a qualified nurse for such position?—*H. M.*

Apply to the manager, P. and O Company, 122, Leadenhall Street, E.C. It is not necessary to be a nurse, it may be an advantage.

*Weak Intellect.*

(186) *W. H. Knighton* may be glad to know of the National Association for Promoting the Welfare of the Feeble Minded (as distinct from idiots and imbeciles). The Secretar , 49, Victoria Street, will give all information respecting the boys' homes.

*Monthly Fees.*

(187) I was engaged to nurse a lady for the middle of February for one month, fee five guineas. The case came off a month earlier. I was not sent for. Can I claim my fee? I had not the engagement in writing, as I went to see the lady by appointment, and refused two other cases to attend.—*Nurse Matthews.*

You are entitled to full fees, but not having had the engagement in writing it is difficult to enforce their payment. Why not write to the lady and ask her to pay them, or whatever sum you deem a fair compensation? It is in cases like this that you would find it advantageous to belong to the Midwives' Institute 12, Buckingham Street, Strand. In making your future engage en s have the terms in writing, and stipulate for a definite fee in case your services were not required.

*Salutations.*

(188) District nurse would like to know whether it is correct for her to bow to any of the doctors she knows when meeting them in the street, or must she wait to see if they salute her first?—*M. M. C.*

There is no definite rule, and a district nurse would spare herself much unpleasantness if she made no claim for social recognition from medical men she only knows professionally.

*The Holt-Ockley System.*

(189) What does the "Holt-Ockley System" mean?—*Nurse E. A. H.*

The Holt Ockley is a system by which respectable, tidy women are given a short training (about six months), and are then employed as cottage attendants to the sick. They are expected to do all the necessary household work, excepting the washing. See "The Ockley Scheme" in "Mirror," October 5th, 1895.

*Hoblyn's Dictionary.*

(190) Would you kindly tell me where I can get Hoblyn's Dictionary, and the price of it?—*M. P.*

Of Messrs. Whittaker and Co., Paternoster Square. The price is 10s. 6d.

*Fees at the Manchester Maternity Hospital.*

(191) We are informed that the fee of £15 15s. at this hospital is inclusive of everything excepting laundry for resident pupils taking the midwifery course. The fee of £5 5s. is that paid by non-resident pupils lodging with and receiving their practical instruction from one of the district midwives attached to the hospital. Consequently our reply (No. 171) was erroneous, as also is the statement with regard to the fees at this hospital in Miss H. Morten's "How to Become a Nurse."

*How to obtain the L.O.S.*

(192) What course should I take to obtain the L.O.S. certificate, and what fees are attached to it? I have plenty of experience, but am not certificated.—*Nurse S. P.*

If you can obtain a medical man's certificate that you have attended 25 cases you need only take up the theory. Perhaps you might learn this by correspondence, in which case Miss Lamport, 52, St. John's Wood Road, N.W., will give you particulars as to terms for coaching and examination. You should have sent your full name.

*Experts in the Hospital Kitchen.*

(193) Will you kindly tell me whether diplomees in cookery are to any extent employed as kitchen matrons or superintendents in hospitals?—*Diplomee.*

We have not heard of them. Perhaps your query will bring forth some useful information on the subject.

